# Effectiveness of Two Different Behavior Modification Techniques for Anxiety Reduction in Children

**DOI:** 10.7759/cureus.28141

**Published:** 2022-08-18

**Authors:** Nilima R Thosar, Sphurti P Bane, Pranjali V Deulkar, Meghana A Deshpande, Suruchi Gupta

**Affiliations:** 1 Pediatric and Preventive Dentistry, Sharad Pawar Dental College and Hospital, Datta Meghe Institute of Medical Sciences (Deemed to be University), Wardha, IND; 2 Pediatric and Preventive Dentistry, Private Practitioner, Mumbai, IND; 3 Pediatric and Preventive Dentistry, Private Practitioner, Nagpur, IND; 4 Pediatric and Preventive Dentistry, Private Practitioner, Delhi, IND; 5 Pediatric and Preventive Dentistry, Private Practitioner, Varanasi, IND

**Keywords:** behavior management, pediatric dentistry, magic tricks, audiovisual distraction, anxiety

## Abstract

Background

Dental anxiety has been a major concern for dentists while operating children. For a smooth, uneventful treatment, operators must incorporate various behavior management techniques in their practice. The incorporation of magic tricks as behavior management techniques has been used earlier by physicians and nurses to reduce pre-operative anxiety in hospitals. This study aimed to compare the impacts of magic tricks on reducing dental anxiety in children.

Material and methods

Patients aged four to 11 years were eligible for inclusion. The study comprised two groups of 15 children each. During the first visit, children weren’t subjected to any behavior management. Behavior management aids (magic tricks and audiovisuals) were used during the second visit. Hemodynamic parameters along with an anxiety scale were used to assess anxiety in children before, during, and after treatment procedures. Venham’s picture test and modified visual analog scale were also used to assess the anxiety.

Results

A reduction in anxiety was seen in both groups after behavior management was used. The hemodynamic parameters like blood pressure and pulse rate were seen to decrease during the second visit, while the oxygen saturation was seen to increase.

Conclusion

The study demonstrates that magic trick along with audiovisual aids was effective in controlling dental anxiety. Thus, magic tricks can be used in dental practice as a behavior management aid for children to facilitate cooperative behavior. Reducing a child's dental anxiety through various different magic trick aids could be a potential behavior management modality that needs further research.

## Introduction

A child's approach and acceptance towards dental treatment depend on the first dental experience. For any dental treatment to be successful, getting into the good graces of children and gaining their confidence to render cooperation are of utmost importance. Dental anxiety has been a matter of concern for many years and can be defined as a non-specific feeling of apprehension, worry, uneasiness, or dread, the source of which may be vague or unknown [1]. In the dental environment, a child rates the sight, sound, and sensation of the airotor along with the syringe as the most fear-eliciting stimuli [2,3]. Behavioral problems arise when the child is unable to cope with the dental setup which could provoke fear.

Behavior management techniques are procedures intended to improvise a child's coping skills, so that compliance and acceptance towards dental treatment are attained, eventually taming the child's preconceived notion about any dental situation. An array of behavioral guidance techniques are available to manage anxiety in children [4]. However, distraction-based non-aversive behavior management techniques seem to have better acceptance by parents. Distraction alters a child’s distress by disrupting attention and helping in the completion of the dental procedure with minimal stress [5,6].

Audio-visual method is a known behavior guidance aid based on the distraction principle which has helped in reducing anxiety and achieving cooperation in uncooperative children during their dental visits. Nevertheless, there are certain downsides pertaining to using this modality. These visual aids usually discourage the child and operator interaction and induce addiction among the children apart from the cost required for its setup. Magic tricks are interactive humor and illusion-based therapy which distracts children from their medical anxieties. These therapies can include children enjoying the interactive magic shows or learning how to perform these tricks or both. According to medical literature, the use of magic tricks or magic sessions to reduce anxiety in children has been appreciated in the past [7-9]. Thus, the aim of our study was to compare whether magic trick was as efficient as audio-visual behavior guidance aid in reducing child’s anxiety during dental procedures.

## Materials and methods

The study design and methodology were approved by the Institutional Ethical Committee of Datta Meghe Institute of Medical Sciences (Deemed to be University) with approval number DMIMS(DU)/IEC/2019/7907. This was an observational study conducted in the Department of Pediatric and Preventive Dentistry, Sharad Pawar Dental College, Datta Meghe Institute of Medical Sciences (Deemed to be University) for a period of four months from April 2019 to July 2019. The study protocol was explained to the parents/guardians of the children to be included in the study and written informed consent was obtained.

Children of the age group of four to 11 years with Frankl behavior ratings 2 and 3 and ASA grades I and II on their first dental visit were included in the study. Children who were cooperative, below four years of age, undergoing emergency treatment, or had special health care needs were excluded from the study. Based on the previous literature and using the sample size formula, a total of 30 children were made part of the study, who were randomly divided by lottery method into following two groups: the magic trick group (group MT) and the audio-visual group (group AV), with 15 children in each group.

On their first visit, patients of both groups were subjected to oral prophylaxis and only communication was used as a behavior guidance technique. During their second visit, single tooth glass ionomer cement restorations were done in children under a rubber dam without administration of local anesthesia. The patients were subjected to the allotted behavior guidance aid during the complete procedure. The patients of group MT were subjected to the magic thumb as behavior guidance aid while group AV was subjected to their favorite cartoon on a mobile screen that was mounted on the dental chair. The same dental assistant performed the magic tricks and started the audiovisuals in order to avoid bias and maintain standardization. The dental assistant was positioned on the left side of the patient for better visualization and less interference in the treatment procedure. She performed the "magic thumb" trick on group MT children during the procedure. For group AV, she enquired and played the patient's favorite cartoon on the mounted mobile screen.

Hemodynamic parameters such as pulse rate and oxygen saturation were assessed with the help of a pulse oximeter at three stages (pre-operatively, intra-operatively, and post-operatively) to evaluate the child’s anxiety during both their dental visits. Venham’s Picture test and modified visual analog scale were used post-operatively to measure anxiety levels, where the child was asked to point towards the face which he/she felt most closely depicted their feeling.

Statistical analysis

The software used for statistical analysis was Statistical Package for Social Sciences (SPSS) version 24.0 (Chicago, IL: IBM Corporation), and a p-value of <0.05 was considered significant. The data used for the analysis of the results were represented as mean and standard deviation. Chi-square test is a qualitative analysis used to analyze the anxiety scales. Independent t-test and ANOVA test were used to analyze the hemodynamic parameters.

## Results

Table 1 shows that on the second visit pre-operative stage, the mean difference in the pulse rate was non-significant. However, a significant difference was seen in mean pulse rate during intra-operative and post-operative stages on the second dental visit for group MT (p=0.042 and 0.016, respectively), and a similar significant difference was seen even on the second visit during intra-operative stage for group AV (p=0.012) (Table 2).

**Table 1:** Comparison of vital signs (pulse per minute) in group MT between first and second visit. *P-value is significant. NS: non-significant; MT: magic trick

Parameters		First visit	Second visit	p-Value
Pulse per minute	Pre-operatively	98.26±11.08	101.13±8.04	0.455 (NS)
	Intra-operatively	104.13±5.82	96.93±11.73	0.042*
	Post-operatively	102.93±6.89	94.86±10.09	0.016*

**Table 2:** Comparison of vital signs (pulse per minute) in group AV between first and second visit. *P-value is significant. NS: non-significant; AV: audio-visual

Parameters		First visit	Second visit	p-Value
Pulse per minute	Pre-operatively	98.80±7.94	99.26±9.63	0.869 (NS)
	Intra-operatively	102.40±11.05	96.93±11.73	0.012*
	Post-operatively	99.46±10.21	94.86±10.09	0.058 (NS)

The mean oxygen saturation levels in group MT have no significant change during the pre- and intra-operative stages on the second visit. A statistically significant difference in the oxygen saturation levels was seen during the second visit post-operative stage (p=0.019) in group MT (Table 3). However, group AV had a significant difference during the intra-operative stage (p=0.003) indicating low anxiety levels during their second visit (Table 4).

**Table 3:** Comparison of vital signs (oxygen saturation in percentage) in group MT between first and second visit. *P-value is significant. NS: non-significant; MT: magic trick

Parameters		First visit	Second visit	p-Value
Oxygen saturation in percentage	Pre-operatively	98.46±0.91	98.40±0.63	0.719 (NS)
	Intra-operatively	98.06±1.16	98.53±1.55	0.110 (NS)
	Post-operatively	98.46±0.63	98.13±0.63	0.019*

**Table 4:** Comparison of vital signs (oxygen saturation in percentage) in group AV between first and second visit. *P-value is significant. NS: non-significant; AV: audio-visual

Parameters		First visit	Second visit	p-Value
Oxygen saturation in percentage	Pre-operatively	98.53±0.51	98.26±0.70	0.104 (NS)
	Intra-operatively	98.13±0.63	96.86±0.99	0.003*
	Post-operatively	98.00±1.06	98.46±0.74	0.204 (NS)

All the values mentioned in Tables 1-4 indicated low anxiety levels in both groups when compared to their first dental visit. The pulse rate during the second visit of both the groups was compared and showed that children in group AV were more relaxed than in group MT. The results were statistically significant intra-operatively and post-operatively (p=0.042 and p=0.016, respectively) (Table 5).

**Table 5:** Comparison of vital signs (pulse per minute) during second visit between group MT and group AV. *P-value is significant. AV: audio-visual; MT: magic trick

Parameters		Group MT	Group AV	p-Value
Pulse per minute	Pre-operatively	101.13±8.04	99.26±9.63	0.569 (NS)
	Intra-operatively	96.93±11.73	96.93±11.73	0.042*
	Post-operatively	94.86±10.09	94.86±10.09	0.016*

Similarly, the intra-operative oxygen saturation levels for group MT and AV were 98.53±1.55 and 96.86±0.99, respectively indicating a statistically significant difference in oxygen saturation levels intra-operatively in group AV (p=0.002). The post-operative levels were non-significant (0.199) (Table 6).

**Table 6:** Comparison of vital signs (oxygen saturation in percentage) during second visit between group MT and group AV. *P-value is significant. NS: non-significant; AV: audio-visual; MT: magic trick

Parameters		Group MT	Group AV	p-Value
Oxygen saturation in percentage	Pre-operatively	98.40±0.63	98.26±0.70	0.590 (NS)
	Intra-operatively	98.53±1.55	96.86±0.99	0.002*
	Post-operatively	98.13±0.63	98.46±0.74	0.199 (NS)

A statistically significant difference between the mean Venham’s Picture test and modified visual analog scale scores was obtained on the second dental visit in both group MT (p=0.006 and 0.009) and group AV (p=0.000 and 0.001), indicating a greater reduction in dental anxiety in these two groups (Table 7). Statistically, a significant difference was seen with Venham’s Picture test scores for the second visit during intergroup comparison in group AV (p=0.035) (Table 8).

**Table 7:** Comparison of dental anxiety scores between first and second visit in both groups. MT: magic trick; AV: audio-visual; MVARS: modified Venham’s clinical ratings of anxiety and cooperative behavior scale

Group	Dental anxiety scale	Score in first visit	Score in second visit	p-Value
Group MT	Venham’s picture test	5.06±0.88	4.00±0.84	0.006
	MVARS	1.33±0.48	0.93±0.25	0.009
Group AV	Venham’s picture test	5.33±1.17	3.26±0.96	0.000
	MVARS	1.40±0.50	0.73±0.45	0.001

**Table 8:** Comparison of dental anxiety scores during second visit between both groups. MT: magic trick; AV: audio-visual; MVARS: modified Venham’s clinical ratings of anxiety and cooperative behavior scale

Dental anxiety scale	Group MT	Group AV	p-Value
Venham's Picture test	4.00±0.84	3.26±0.96	0.035
MVARS	0.93±0.25	0.73±0.45	0.152

## Discussion

Communication is the primary strategy of behavior management. A meticulous understanding of the child’s cognitive development and vocabulary is necessary to effectively communicate with the child. Every child cannot express his/her fears and anxieties and thus communication is hampered. Younger children find it difficult to perceive an idea for which they have no prior experience and to understand the dentist's frame of reference [5,10].

Managing a child’s behavior is a vital part of pediatric practice. Behavior guidance modalities have been used by pediatric dentists to establish communication, alleviate fear and anxiety, and build a trusting relationship between dentist, child, and parent. This helps to promote the child’s behavior and develop a positive attitude towards one’s oral healthcare. McCaul and Mallot through their theory stated that a patient’s pain perception can be decreased if he is distracted from an unpleasant stimulus. Neurophysiological studies have confirmed the importance of distraction to lower levels of pain and anxiety, as there is a direct association between pain perception and the amount of attention a child pays to the unpleasant stimulus [11].

Based on this distraction principle, audiovisual aid intervenes in the child’s attention and ensures a positive experience during the treatment [11]. The results noted in the present study were parallel to a study conducted by Prabhakar et al. in 2007 where audiovisual aid was found to be more efficient than an audio distraction in reducing a child’s dental anxiety during various dental visits [12]. Literature suggests that audiovisual aid on comparing with similar non-pharmacological behavior management methods, such as music therapy, playing mobile dental games, and the tell-play-do technique effectively minimized dental anxiety along with enhancing cooperative behavior as seen in the present study. A study by Niharika et al. in 2018 reported that the audiovisual method has a better effect on distracting the children than the tell-show-do technique [11]. Shah et al. in 2018 incorporated the tell-play-do behavior modality into their practice and found its effectiveness in managing children during the dental procedure [13].

In the early 1980s, the incorporation of magic tricks as a therapeutic intervention was used to address psychological issues in children. Magic tricks were used by health professionals to enhance the self-esteem of patients (Howard et al.), and are also helpful in pediatric oncology patients to decrease their anxiety prior to needle insertion (Frankenfield et al.). This modality was commonly used by anesthetists before intubating pediatric patients (Vagnoli et al.) and in pediatric wards (Fisher et al.) [14]. Magic tricks, when used as a behavior guidance technique in the current study, showed a reduction in the mean pulse rate with statistically significant differences during intra-operative and post-operative stages (p=0.042 and 0.016, respectively) and with oxygen saturation levels post-operatively (p=0.019) during the second visit. The results also showed a statistical reduction in Venham’s Picture test and modified visual analog scale (p=0.006 and 0.009, respectively). Similar anxiety reduction was observed in pediatric patients when magic therapy was given and evaluated using Venham’s Pictures test [8]. Also, Alparslan and Bozkurt found that the presence of medical clowns in hospitals reduces the child’s anxiety [15]. The reduction in anxiety can be attributed to mainly the distraction principle.

The right hemisphere of the human brain is characterized by imagination, non-verbal, art, and music-related skills. It is also more developed in children and magic tricks will directly address this part of the human brain [9,16,17]. Thus, a reduction of anxiety in children was seen in the magic trick group during their second visit when compared to their first visit. But when both the groups were compared during their second visit, audiovisual aid had a significant difference in mean pulse rate and oxygen saturation levels during intra-operative procedures (p=0.042 and 0.002, respectively). Audiovisual aids can be played throughout the procedure thus engaging the child completely, whereas the effectiveness of a magic trick is correlated to the visual attention of the child at the exact moment when the magic trick is performed [18]. The effect of magic trick on pre-operative intergroup comparison was found to be statistically insignificant (p=0.569 and 0.590). Similar results were obtained by the only study pertaining to magic tricks being used to reduce anxiety in the dental operatory. Peretz et al. in 2005 demonstrated that magic trick was able to facilitate cooperative behavior in strong-willed children (>90%), and the operators were even able to take radiographs when used pre-operatively (p=0.018) along with significant difference in the time required for the child to sit on the dental chair (p=0.015) [16].

Anxiety reduction indicators used in our study were mainly pulse rate and oxygen saturation levels, as it has been observed that these hemodynamic changes are a direct measure of physiological arousal which increases in values during stress. The Venham’s Picture scale and modified visual analog scale are projective, psychometric, and self-measure tests used to evaluate the child’s anxiety. All these parameters are non-invasive and easy for the operator to measure and note the values.

Learning a basic magic trick can be easy and does not require any special skills and knowledge. Based on the cognitive development of children, dentists can use these distraction techniques consistent with the receiver’s intellectual development. However, there were certain limitations to the present study. The children weren’t divided according to their age to evaluate which behavior guidance aid facilitates a certain age group. Further studies can be undertaken to evaluate the age group where magic tricks as behavior management can be effective.

## Conclusions

Audiovisual aids are commonly used and proven to reduce dental anxiety in children. Magic trick, a revisited behavior guidance aid in the medical literature has proved to be equally effective as audiovisual aid for anxiety reduction in children during dental treatment. Dentists should incorporate magic tricks in their behavior management modalities.
